# Systemic Lymphadenectomy Cannot Be Recommended for Low-Risk Corpus Cancer

**DOI:** 10.1155/2010/490219

**Published:** 2010-02-04

**Authors:** Takao Hidaka, Akitoshi Nakashima, Tomoko Shima, Toru Hasegawa, Shigeru Saito

**Affiliations:** Department of Obstetrics and Gynecology, Faculty of Medicine, University of Toyama, Toyama 930-0194, Japan

## Abstract

*Objective*. The objective of this study is to ascertain whether omission of lymphadenectomy could be possible when uterine corpus cancer is considered low-risk based on intraoperative pathologic indicators. *Patient and Methods*. Between 1998 and 2007, a total of 83 patients with low risk corpus cancer (endometrioid type, grade 1 or 2, myometrial invasion ≦50%, and no intraoperative evidence of macroscopic extrauterine spread, including pelvic and paraaortic lymph node swelling and adnexal metastasis) underwent the total abdominal hysterectomy and bilateral salpingo-oophorectomy without lymphadenectomy. A retrospective review of the medical records was performed, and the disease-free survival (DFS), overall survival (OS), peri- and postoperative morbidities and complications were evaluated. *Results*. The 5-year DFS rates and the 5-year OS rates were 97.6% and 98.8%, respectively. No patient presented postoperative leg lymphedema and deep venous thrombosis. *Conclusion*. Omission of lymphadenectomy did not worsen the DFS or OS. The present findings suggest that systemic lymphadenectomy could be omitted in low-risk endometrial carcinoma.

## 1. Introduction

Endometrial cancer is the most frequently occurring gynecologic malignancy. It accounts for 6% of all cancers in women, and causes approximately 42,000 deaths annually, which represents 3% of cancer deaths in women in the United States [1]. Most of the cancers are detected at an early stage by common symptom such as postmenopausal bleeding, with the tumor confined to the uterine corpus, so the prognosis is generally favorable and surgery alone may result in a cure. The five-year survival rate for stage IA or IB disease is reported to be over 90% [2, 3].

The International Federation of Gynecology and Obstetrics (FIGO) recommended in 1988 that adequate surgical staging requires a total abdominal hysterectomy, bilateral salpingo-oophorectomy (TAH-BSO) including pelvic and paraaortic lymphadenectomy [4], and according to this recommendation, some surgeons believe that lymphadenectomy should be performed in all cases to enable the accurate staging and to assess the necessity for postoperative treatment. However, there are some risks to lymphadenectomy such as postoperative deep vein thrombosis or leg lymphedema which may impair the patients' quality of life. Mariani et al. reported that low-risk corpus cancer (endometrioid type, grade 1 or 2 tumor, myometrial invasion ≦50%, and no intraoperative evidence of macroscopic extrauterine spread) could be treated optimally with hysterectomy only [5]. We retrospectively reviewed the cases of low-risk corpus cancer, which were treated in our hospital, and clarified that lymphadenectomy did not provide a significant survival advantage, and increased peri- and postoperative morbidities and complications [6]. According to these results, since 1998, lymphadenectomy have been omitted in low-risk corpus cancer in our hospital. We retrospectively reviewed these cases and evaluated whether omission of lymphadenectomy for low-risk corpus cancer worsen the disease-free survival (DFS), overall survival (OS), and avoid peri- and postoperative morbidities and complications.

## 2. Patients and Methods

Eighty-three patients (median age: 55 years, range: 27–80 years) with endometrioid corpus cancer, grade 1 or 2 tumor, myometrial invasion ≦50%, and no intraoperative evidence of macroscopic extrauterine spread, including pelvic and paraaortic lymph node swelling and adnexal metastasis, were treated surgically at the Department of Obstetrics and Gynecology of University of Toyama during the period 1998 to 2007. In all these cases, we preoperatively assessed whether endometrial cancer is considered low-risk (myometrial invasion ≦50%, no lymphadenopathy and grade 1 or 2 endometrioid corpus cancer), using computed tomography, magnetic resonance imaging, glucose analog [18F]-fluoro-2-deoxy-D-glucose positron emission tomography and endometrial biopsy.

All patients routinely underwent TAH-BSO without lymphadenectomy. If the depth of myometrial invasion was determined to be >50%, or the tumor was classified grade 3 endometrioid corpus cancer based on intraoperative frozen-section analysis, we performed systemic lymphadenectomy. In cases which lymph nodes were enlarged or suspicious, we performed selective lymph node sampling. Non-endometrioid histologic types such as serous or clear cell type and grade 3 tumor were excluded from this study, since all of these patients underwent TAH-BSO with lymphadenectomy because of their poor prognosis. After operation, patients were seen every month for one year, and every 3 months thereafter for 120 months.

We performed a retrospective review of the medical records, and the disease-free survival (DFS; the interval between the date of operation and the date of recurrence of disease), overall survival (OS; the interval between the date of operation and the date of death), and peri-operative morbidities including operative time, estimated blood loss during operation, and the percentage of patients requiring transfusion were evaluated. We also estimated the incidence of postoperative complications such as leg lymphedema and deep vein thrombosis diagnosed by nuclear venography. The DFS and OS curves were estimated using the Kaplan-Meier method.

## 3. Results

Patients' characteristics are shown in Table 1. The median age of the patients was 55 years (range, 27–80 years). The distribution of FIGO surgical stage was Ia, 32; Ib, 43; Ic, 3, IIIa, 5; and 8 cases (9.6%) were upstaged postoperatively. Pelvic lymph node sampling was performed in 7 cases, which were diagnosed with negative nodes. Adjuvant chemotherapy consisting of intravenous paclitaxel (180 mg/m^2^) and carboplatin (AUC 5) was administered to 9 upstaged, upgraded or lymphvascular space involvement-positive cases. No patients received adjuvant radiotherapy. The median followup period was 72 months (range, 4–120 months).

Characteristics of the histopathological prognostic features are shown in Table 2. The distribution of histological grade was grade 1, 72; grade 2, 10; grade 3, 1; and 1 case (1.2%) was upgraded postoperatively. Depth of myometrial invasion was >50% in 3 cases. Lymphvascular space involvement was observed in 5 cases. Positive peritoneal cytology was observed in 5 case. Thirty-nine patients had tumor diameter ≧20 mm. 

**Figure 1:**
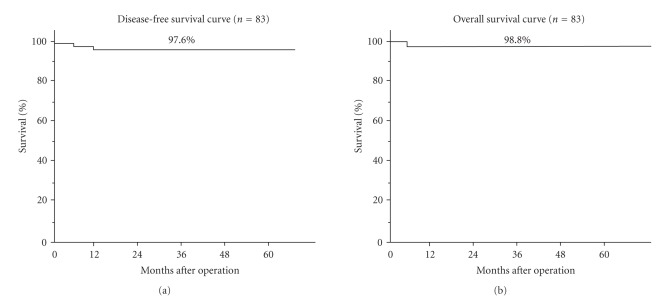
Survival curves.

The DFS curve and the OS curve are shown in Figure 1 (left; the DFS curve, right: the OS curve). The 5-year DFS rates and the 5-year OS rates were 97.6% and 98.8%, respectively.

Peri- and postoperative morbidities and complications are shown in Table 3. The mean operative time was 129 ± 28 minutes. The mean estimated blood loss during operation was 244 ± 192 mL, and the percentage of transfusion requirement was 2.4%. No patient presented with postoperative leg lymphedema or postoperative deep venous thrombosis.

## 4. Discussion

In 1988, FIGO recommended a systemic surgical staging system for corpus cancer [4]. According to this recommendation, many gynecologic oncologists believe that systemic surgical staging including pelvic and paraaortic lymphadenectomy is the best surgical treatment to achieve a good prognosis for corpus cancer patients. However, controversy has persisited regarding the need for lymphadenectomy [5–9]. There is no doubt that surgical staging is more accurate than clinical staging, and there are some cases which need upstaging to higher stages after surgery [10–12]. However, Morrow et al. reported that only 18 (2%) out of 895 patients had positive pelvic lymph nodes in the abscence of operative findings by palpation [13]. Creasman et al. demonstrated by multivariate analysis that in clinical stage I patients with grade 1 or 2 and depth of invasion within the middle 1/3, the incidence of pelvic lymph node metastases was only 3.6% [7]. Chi et al. showed that the incidence of pelvic lymph node metastases in low-risk corpus cancer (grade; 1, 2, and depth of myometrial invasion; none or inner half) was 5.3% (14/162) [14]. As for grading, Ben-Shachar et al. reported that only 6 (3.3%) of 181 patients preoperatively diagnosed with grade 1 disease by biopsy were upgraded after surgical staging [15]. In this study, 8 of the 83 were upstaged or upgraded after surgical staging.

Regarding the role of lymphadenectomy on prognosis, Mariani et al. reported that patients who had grade 1 or 2 endometrioid corpus cancer with greatest surface dimension ≦2 cm, myometrial invasion ≦50%, and no intraoperative evidence of macroscopic disease could be treated optimally with hysterectomy only with favorable prognosis of up to 97% for 5-year overall cancer-related survival [5]. Furthermore, Trimble et al. demonstrated that the 5-year relative survival for 6,363 women with stage I endometrial cancer who did not undergo lymph node sampling was 98%, compared to 96% for 2,831 women who did undergo lymph node sampling at the time of hysterectomy with a no significant difference, and concluded that lymph node sampling did not appear to convey survival benefit, especially in stage I, grade 1 or 2, endometrial cancer by subgroup analysis [9] Recently, Kitchener et al. compared the standard surgery group (hysterectomy and bilateral salpingo-oophorectomy, peritoneal washings, and palpation of para-aortic nodes; *n* = 704) and the lymphadenectomy group (standard surgery plus lymphadenectomy; *n* = 704) for histolgically proven endometrial carcinoma thought preoperatively to be confined to the corpus in a randomised study and showed that the hazard ratio (HR) for overall survival was 1.04 (0.74–1.45; *P* = .83) and HR for recurrence-free survival was 1.25 (0.93–1.66; *P* = .14), both in favour of standard surgery, and concluded that there was no evidence of benefit in terms of overall or recurrence-free survival for pelvic lymphadenectomy with early endometrial cancer, and that pelvic lymphadenectomy cannot be recommended as routine procedure for therapeutic purposes outside of clinical trials [16]. Panici et al. also, compared the pelvic systematic lymphadenectomy arm (*n* = 264) and no lymphadenectomy arm (*n* = 250) for preoperative FIGO stage I endometrial carcinoma and showed that the 5-year disease-free and overall survival rates were similar between the two arms (81.0% and 85.9% in the lymphadenectomy arm and 81.7% and 90.0% in the no-lymphadenectomy arm) [17]. In summary, omission of complete lymphadenectomy is possible in selected cases in which the risk of lymph node spread is low, in other words, low-risk cancer. The definition of low-risk in corpus cancer at the operation is controversial; however, taking many reports into consideration, we regard grade 1 or 2 endometrioid corpus cancer with myometrial invasion ≦50%, and no intraoperative evidence of macroscopic disease as low-risk [5–9, 18, 19].

There were 8 upstaged patients on final pathology who were thought low-risk on pre- and intraoperative evaluation. This result means that there is some limitation about the accuracy of pre- and intraoperative evaluation of myometrial invasion and tumor grade. Montalto et al. reported that accuracy of intraoperative frozen section diagnosis for grade of differentiation and depth of myometrial invasion were 84.3% and 94.3%, respectively [20]. For those upgraded, upstaged or lymphvascular space involvement-positive cases, as well as, non-endometrioid adenocarcinoma such as uterine papillary serous carcinoma, which is clinically aggressive, adjuvant treatment should be considered. Recently, paclitaxel has been shown to be effective against advanced and recurrent endometrial carcinoma [21–25]. Therefore, we added systemic chemotherapy (a paclitaxel/carboplatin regimen) as adjuvant treatment for upgraded, upstaged or lymphvascular space involvement-positive cases.

Several investigators have reported that addition of lymphadenectomy to TAH-BSO increases the risk of complications and morbidities such as more blood transfusions, longer hospital stay, lymphedema, gastrointestinal injury, and the development of lymphocysts [26–28]. Framarino et al. reported that addition of pelvic and paraaortic lymphadenectomy to TAH-BSO significantly increased mean operative time, mean estimated blood loss, and postoperative hospital stay compared to TAH-BSO alone, without improving mortality [28]. Panici et al. showed that postoperative complications occurred statistically significantly more frequently in patients who had received pelvic systematic lymphadenectomy (81/264; 30.7% in the lymphadenectomy arm and 34/250; 13.6% in the no-lymphadenectomy arm, *P* = .001) [17]. Also in our previous study, mean operative time, mean estimated blood loss during operation, the percentage of cases requiring transfusion, and the incidence of leg lymphedema were significantly (*P* < .001) increased by addition of lymphadenectomy [6]. In any case, postoperative complications are expected from the surgical procedure itself, and addition of lymphadenctomy may increase the incidence of those complication, especially in corpus cancer patients, many of whom have morbidities such as hypertension, obesity, diabetes mellitus, and older age [29, 30].

## 5. Conclusion

A clinically important goal of surgical treatment including lymphadenectomy for low-risk corpus cancer patients is not only to determine the extent of disease and an accurate prognosis, but also to obtain a favorable prognosis without causing any complications. Our data demonstrate that omission of lymphadenectomy did not worsen the disease-free or overall survival, and as a result, peri- and postoperative morbidities and complications could be avoided. The present findings suggest that systemic lymphadenectomy could be omitted in low-risk endometrial carcinoma. These results should be confirmed in future prospective large-scale randomized clinical trials.

## Figures and Tables

**Table 1:** Patients characteristics (*n* = 83).

Age (years)	
Mean ± SD	56.2 ± 12.1
Median	55
Range	27–80
WHO* performance status, no.	
0	59
1	14
>2	0
FIGO** surgical stage, no.	
Ia	32
Ib	43
Ic	3
IIIa	5
Adjuvant chemotherapy, no.	
None	74
Paclitaxel/carboplatin^§^	9
Follow up interval (months)	
Median (range)	72 (4–120)

WHO*: World Health Organization, FIGO**: International Federation of Gynecology and Obstetrics Paclitaxel/carboplatin ^§^: Paclitaxel (180 mg/m^2^) and carboplatin (area under the curve; AUC 5).

**Table 2:** Characteristics of the histopathological prognostic features (*n* = 83).

Histological grade, no.	
G1	72
G2	10
G3	1
Depth of myometrial invasion >50%, no.	
None	32
≦50%	48
>50%	3
Lymphvascular space involvement, no.	
Yes	5
No	78
Peritoneal cytology, no.	
Positive	5
Negative	78
Tumor diameter, no.	
<20 mm	44
≧20 mm	39

**Table 3:** Peri- and postoperative morbidities and complications.

Peri- and postoperative factors	
Operative time* (min)	129 ± 28
Estimated blood loss during operation* (mL)	244 ± 192
Transfusion requirement, no.	2
Postoperative leg lymphedema (≧grade 2, NCI-CTC ver. 2.0), no.	0
Postoperative deep vein thrombosis, no.	0

*The values were expressed as the mean ± SD.
